# Relations of Science to Speculation

**Published:** 1881-09

**Authors:** J. W. Dawson


### RELATIONS OF SCIENCE TO SPECULATION.

A LECTURE BY J. W. DAWSON, LL.D.

DO WE really exist? If we do, what is the thing called life?

If we ask, what is science in relation to nature, we have before us all that men have observed of the workings and objects of nature and the deductions therefrom. But added to this is something of another sort, sometimes called philosophy, which is really a mass of material, the growth of the thoughts of the times, and this is that troublesome commodity—modern speculation. Evolutionists need the less complain of this view, as it is the natural outgrowth of their theory. It by no means follows that our knowledge of recent discoveries equals the extent of their practical application. Take, for instance, electricity. Its application in a variety of ways is almost general, and yet there are very few things we know less about, either as to its laws or by what it is regulated. There is so much discovery that men are in danger of thinking themselves omniscient, but in reality the most of the accomplishments of science remain mysteries to mankind. The tendency to mad specialties of study, too, causes a great deal of general speculation, covering the whole field to be made, from a point of view that is really restricted. Then there has sprung up a demand for sensational science just as there has sprung up a demand for sensational fiction, and if it cannot be met by facts in existence, something has to be invented, for the number of half-starved scientific men who are trying to find the secrets of nature is by no means small. Moreover, a great deal of supposed scientific truth is vague and does not mean one-half what is supposed. I don't know to what extent science is to be blamed, but not a few of those who have pushed speculation to its utmost have been literary men, and some of them had studied theology as well. An agnostic, you know, is literally one who doesn't know. As one of them has expressed the idea, "the existence of a God is unthinkable." The agnostic while saying less than the atheist, means scientifically more, and steels himself against argument by saying it cannot be reasoned about at all. In the true sense no scientific man can be an agnostic. There are a few of us, perhaps, who will refuse to accept the creed, "I exist," although there are some who might limit it, and yet our personal existence is a thing most incomprehensible, especially in regard to its beginning and its ending.

We are in a boundless space and time, with the beginning of either a mystery, so that it is scarcely possible for any one to admit their own existence without arousing a vestige of the religious idea. In personal existence is involved the question whether or not the vastly complex bodily organism is the first or only the outer shell. We cannot but admit that the body is vastly more mysterious since science took it up. There is a class of philosophers who think it will simplify matters to consider the individual as a material substance with two sets of properties. If by a material substance is meant a combination of elements there is no such material in existence having the qualities of mind; if it means the whole being, the statement amounts to nothing. We must admit there is something more, and that is life. Nutrition, reproduction, sensation and voluntary motion are its functions, the last two being restricted to animal life. Herbert Spencer says life is "the continuous adjustment of internal and external relations," but that is very vague and only touches the surface. I think myself that life has the same relation to organisms that force has to matter. It is some energy, whether a combination of physical energies, or the same but correlated only with organization. It is all very well to find fault with calling the living organization "a machine," but a machine has to be made by some one and for a purpose, and there is an enormous lot of theism in the idea. Who ever heard of a machine made by nobody for nothing in particular? What is the use in talking about protoplasm as the basis of life when in point of fact protoplasm depends on life as its basis? All the white of an egg is protoplasm, but life is in the embryo cell. Protoplasm may go to make feathers and flesh and tissue, and yet the life that constitutes the chick may be elsewhere. Going further, science fails to correlate the power of the human will with any physical force. It is an energy that operates only on living organs and through them on other things Then comes the question, are we the originators of the world, or did it produce us, or is there a great third Being? If an atom is a vortex, as they say, what is it a vortex of? We talk very learnedly of laws of nature, but they are but the expressions of the controlled motion of things. Behind them lies an insoluble mystery.

In conclusion Mr. Dawson discussed the three theories of Herbert Spencer as to the origin of things, "self-existing," or "self-created," or "created by external agency," and raised a laugh by saying that the possibility of creation by an agency within had never occurred to Mr. Spencer. Mr. Dawson's deduction was that science is not inconsistent with the view of a superhuman [power as explaining the origin, design, and continuance of things.—*Philadelphia Times.*

